# Description of a new species of the genus *Araeopteron* Hampson, 1893 (Erebidae, Boletobiinae) from China, with a new subspecies of *A. nigrizonatum* Hirano, 2021

**DOI:** 10.3897/BDJ.14.e200131

**Published:** 2026-06-29

**Authors:** Chun-Hua Yao, Cheng-De Li, Hui-Lin Han

**Affiliations:** 1 College of Forestry, Northeast Forestry University, Harbin 150040, China, Harbin, China College of Forestry, Northeast Forestry University, Harbin 150040, China Harbin China; 2 Ministry of Education, Key Laboratory of Sustainable Forest Ecosystem Management, Northeast Forestry University, Harbin 150040, China, Harbin, China Ministry of Education, Key Laboratory of Sustainable Forest Ecosystem Management, Northeast Forestry University, Harbin 150040, China Harbin China; 3 Northeast Asia Biodiversity Research Center, Northeast Forestry University, Harbin 150040, China, Harbin, China Northeast Asia Biodiversity Research Center, Northeast Forestry University, Harbin 150040, China Harbin China

**Keywords:** Araeopteronini, *A. nigrizonatum*, *A. zheganensis*, Noctuoidea, Taxonomy

## Abstract

**Background:**

A new species of the genus *Araeopteron* Hampson, 1893 from China: *Araeopteron zheganensis*, sp. nov., collected from Zhejiang and Jiangxi Provinces, as well as a new subspecies, *A. nigrizonatum chinensis*, ssp. nov., recorded from Yunnan, Fujian, Zhejiang and Chongqing.

**New information:**

The study provides illustrations of the adults and genitalia of *A. zheganensis*, sp. nov. and the new subspecies *A. nigrizonatum chinensis*, ssp. nov. and includes comparative analyses with morphologically similar species.

## Introduction

The genus *Araeopteron* Hampson, 1893 was established with *Araeopteron pictale* Hampson, 1893 as its type species. Since its establishment, the taxonomic placement of this genus has undergone considerable changes. In early studies, due to the instability of the classification system of Noctuoidea, several synonymous genera related to *Araeopteron* were proposed, including *Araeopterum* Hampson, 1895, *Essonistis* Meyrick, 1902, *Thelxinoa* Turner, 1902 and *Araeoptera* Hampson, 1910, these names were subsequently being synonymised with *Araeopteron* (*Fibiger and Kononenko 2008*). Meanwhile, *Araeopteron* had been assigned to different subfamilies within Noctuidae sensu lato (Hampson 1910, Inoue 1958, Inoue 1965, Nye 1975, Poole 1989, Kononenko 1990, Kononenko 2003, Kononenko 2005). With the advancement of phylogenetic studies on Noctuoidea and the gradual stabilisation of its classification system, the systematic position of *Araeopteron* has been clarified. It is currently placed in the tribe Araeopteronini of the subfamily Boletobiinae within Erebidae (Fibiger and Lafontaine 2005, Zahiri et al. 2012).

Members of *Araeopteron* are characterised by a set of stable morphological synapomorphies, including small body size, a distinctive wing shape with a narrow fore-wing and triangular hind-wing, as well as specific wing pattern elements and specialised male and female genital structures (Fibiger and Lafontaine 2005). At present, the genus comprises more than 70 species, mainly distributed in tropical and subtropical regions (Turner 1902, Hampson 1907, Hampson 1910, Turner 1925, Turner 1933, Inoue 1958, Inoue 1965, Fibiger and Hacker 2001, Fibiger and Hacker 2002, Hirano 2021) . In China, 13 species have been recorded to date (Wu et al. 2020, Han and Kononenko 2021, Cheng and Han 2022, Jin et al. 2025). Recent taxonomic studies have continued to reveal previously undescribed species and the diversity of this genus remains insufficiently explored in many regions, suggesting that its actual species richness is likely underestimated.

In the present study, two new taxa of *Araeopteron* from southern China are described. This study provides new data for a better understanding of the taxonomy and diversity of the genus.

## Materials and methods

The material examined in this study was collected from southern China using a 220 V/450 W high-pressure mercury lamp for light trapping. All adult specimens were prepared following standard procedures for Lepidoptera, including wing spreading and setting. Genitalia were prepared using conventional dissection techniques. The abdomen was removed and macerated in a 15% potassium hydroxide (KOH) solution. After dissection, the genital structures were separated and the vesica of the aedeagus was everted. The dissected parts were then dehydrated through a graded ethanol series, stained with eosin, cleared in xylene and finally mounted on permanent slides using neutral balsam. Adult habitus photographs were taken using a Nikon Z8 camera. Images of genitalia slides were captured using an AO-HK830-0318 microscope. All images were stacked using Helicon Focus and subsequently processed in Adobe Photoshop 2025 for final optimisation. All specimens examined in this study are deposited in the Insect Collection of Northeast Forestry University, Harbin, China.

**Abbreviations used** :

- **NEFU** – Northeast Forestry University, Harbin, China
- **HT** – Holotype
- **PT** – Paratype
- **YCH** – Slide prepared by Chun-Hua Yao

## Taxon treatments

### Araeopteron

Hampson, 1893

D2B2C8F9-8524-5B40-B06B-21F95BFE0A18

Araeopteron Hampson, 1893; Illustrations of Typical Specimens of Lepidoptera Heterocera in the Collection of the British Museum 9: 33, 136. Type species: *Araeopteron pictale* Hampson, 1893. Type locality: Sri Lanka.
= *Araeopterum* Hampson, 1895; unjustified emendation of *Araeopteron* Hampson, 1893.
= *Essonistis* Meyrick, 1902; Type species: *Essonistis micraeola* Meyrick, 1902. Type locality: Australia.
= *helxinoa* Turner, 1902; Type species: *Thelxinoa epiphracta* Turner, 1902. Type locality: Australia.
= *Araeoptera* Hampson, 1910; unjustified emendation of *Araeopteron* Hampson, 1893.
= *Araeopterella* Dyar, 1914; Type species: *Araeopterella miscidisce* Dyar, 1914. Type locality: Panama; synonym of *Araeopteron* Hampson, 1893.
= *Araeopterella* Fibiger & Hacker, 2001; *Esperiana* 8: 581 (preocc. *Araeopterella* Dyar, 1914); Type species: *Araeopterella sterrhaoides* Fibiger & Hacker; synonym of *Araeopteron* Hampson, 1893.
Araeopteron pictale Hampson, 1893

#### Diagnosis

The genus *Araeopteron* Hampson, 1893 is characterised by the frons with loose or smooth scales; the fore-wing is slender and elongate, with the reniform spot black; the hind-wing is much shorter than the fore-wing, with the outer margin concave below the apex. In the female, a slightly sclerotised patch or conical process bearing long scales is present between the terminal abdominal segment and the papillae anales. In the female genitalia, the signum is mostly conical or in the shape of a cap or a shuttlecock, rounded apically and spinose basally, sometimes large, flattened and lamellar. In the male genitalia, the sacculus is strongly sclerotised, narrow, elongate and flattened, forming the longest portion of the valva; the apex of the sacculus is in the shape of a spoon or a club; the clasper and the dorsal margin of the cucullus are membranous; the tegumen is short and broad, usually moderately to strongly sclerotised and twisted; the vinculum is short and broad; the uncus is long, with a broad junction between the base of the uncus and the apex of the tegumen.

### Araeopteron zheganensis sp. nov.

F5C8AD1F-8305-5028-899C-87343C782648

6ABC1240-C206-4B75-A8CB-8B948E705BF1

#### Materials

- **Type status:** Holotype. **Occurrence:** recordedBy: Zhang et al. leg.; sex: male; lifeStage: adult; occurrenceID: DC19145E-01E3-5E81-BFCE-5C0D9E822E17; **Location:** country: China; stateProvince: Zhejiang; county: Jiangshan; locality: Laofoyan Village; **Event:** eventDate: 5 Ⅶ 2017; **Material Entity:** disposition: in NEFU
- **Type status:** Paratype. **Occurrence:** recordedBy: H.L. Han; sex: male; lifeStage: adult; occurrenceID: EA1057DF-CE1F-5167-9A58-B8DBA2C77099; **Location:** country: China; stateProvince: Jiangxi; county: Jinggangshan; locality: Liujiaping Village; **Event:** eventDate: 3–9 Ⅷ 2021; **Material Entity:** disposition: in NEFU

#### Description

**Adult** (Fig. 1a, b) Wingspan 11–12 mm in male. Head with pale yellow scales; antennae filiform. Patagium, tegula with dense white and pale yellow mixed scales. Legs with light brown scales. Fore-wing yellowish-brown; costal margin with longitudinal black-and-white stripes from base to distal half. Basal band, antemedial line brown, discontinuous; median, postmedial lines parallel, brown, arcuate, thickened at costa. Praeterminal line brown, discontinuous, with patch-like spots; subterminal line brown, indistinct; terminal line brown, dark brown at veins. Reniform spot brown, indistinct. Outer margin with fringe alternating dark and light brown. Hind-wing as fore-wing, transverse lines thickened at inner margin, forming dark brown patches. Abdomen with yellowish-brown to dark brown scales.

**Male genitalia (Fig. 2a)** Uncus slender hook, base broad, tapering to apex, slightly curved, apex sharply pointed. Tegumen conical. Vinculum broad, band-shaped, arcuate. Valva broad, subtriangular, base narrow, strongly expanded distally; costal process subquadrate, with slender setae; digitus robust, spine-like, apex acute, slightly curved posteriorly; sacculus as band-like ridge; sacculus process enlarged, slightly exceeding costa, densely with granular protuberances; clasper minute, hemispherical. Juxta lateral margins smooth; dorsal margin medially raised into tongue-like projection; ventral margin semicircular, medially slightly concave. Saccus truncate, subquadrate. Aedeagus stout, short, cylindrical, uniform in width; vesica membranous; cornuti long, tuft-like, strongly sclerotised, curved.

**Female.** Unknown.

#### Diagnosis

The wing pattern of the new species is similar to that of *A. simaoensis* Jin, Yao & Han, 2025 (Fig. 1c), but it differs in having the fore-wing costal margin with a black-and-white longitudinal stripe extending from the base to the distal half (in *A. simaoensis* absent); reniform stigma is pale yellowish-brown (in *A. simaoensis* black). The male genitalia differ distinctly from those of *A. simaoensis* (Fig. 2b); uncus is short and slightly curved (in *A. simaoensis* long and strongly curved); costal process is subquadrate (in *A. simaoensis* semicircular); digitus is stout, short and nearly straight (in *A. simaoensis* slender and S-shaped curved); saccus is truncate and quadrate (in *A. simaoensis* arcuate); aedeagus is of uniform width (in *A. simaoensis* narrow medially and thickened at both ends); cornuti are distinct and formed as long, tufted setae (in *A. simaoensis* indistinct, represented by granular projections).

#### Etymology

The specific epithet *zheganensis* is formed by combining “Zhe” and “Gan” the conventional abbreviations of Zhejiang and Jiangxi provinces, respectively, to indicate the provenance of the type specimens.

#### Distribution

China (Zhejiang, Jiangxi)

#### Biology

This species was collected from montane forest regions in eastern China. Adults were obtained by light trapping during July–August, indicating pronounced phototactic behaviour and suggesting that its peak activity period occurs in summer. Its host plants and other ecological characteristics remain unknown.

### Araeopteron nigrizonatum chinensis n.

3D05373B-125E-5B9A-8BDD-06D2877DC520

E3FEC5E3-CC4A-43D1-B4AF-51A636E4A4CC

#### Materials

- **Type status:** Holotype. **Occurrence:** recordedBy: Z.G. Zhang et al.; sex: male; lifeStage: adult; occurrenceID: D5D595ED-5683-5D1B-B8FF-FB27785C20FA; **Location:** country: China; stateProvince: Zhejiang; county: Quzhou; locality: Gutianshan National Nature Reserve; **Event:** eventDate: 7 Ⅶ 2017; **Material Entity:** disposition: in NEFU
- **Type status:** Paratype. **Occurrence:** recordedBy: Z.G. Zhang et al.; sex: male; lifeStage: adult; occurrenceID: E1F9D5DF-9452-5519-851F-B998A45567B8; **Location:** country: China; stateProvince: Chongqing; county: Jiangjin; locality: Mt. Simian town Dawopu area; **Event:** eventDate: 29 Ⅶ–2 Ⅷ 2020; **Material Entity:** disposition: in NEFU

#### Description

**Adult** (Fig. 1d, e) Wingspan 11–13 mm. Head yellowish-brown with black stripes; antennae filiform, dark brown basally, yellowish-brown distally. Fore-wing costa from base to middle with two black stripes with white longitudinal lines; ground colour pale yellow; antemedial line brown, wavy; median, postmedial lines greyish-brown, patchy, discontinuous. Postmedial line externally with greyish-white band. Terminal line dark brown, discontinuous. Fringe dense, dark. Hind-wing transverse line blackish-brown internally, pale yellow externally. Terminal line discontinuous, alternating dark brown and orange-yellow.

**Male genitalia** (Fig. 2c) Uncus slender, apex acute. Tegumen subconical, lateral margins arcuate. Valva elongate, apex bluntly rounded, with spoon-shaped concavity; costal process medially slightly raised. Juxta bottle-shaped, lower part broadly expanded, arcuate, medially constricted, upper part slightly expanded. Saccus broadly U-shaped. Aedeagus slender, medially constricted, caecum slightly swollen. Carinal hook small, finely serrate; vesica with small spine-like cornuti.

**Female.** (Fig. 3a) Papillae anales broad. Apophyses anteriores and posteriores equal in length. Ostium bursae sac-like; ductus bursae straight and slightly sclerotised. Corpus bursae with tubular part slender, elongate; globular part elongate-elliptical. Signum brush-shaped, compact and clustered, opposite side densely with punctate protuberances.

#### Diagnosis

The new subspecies is very similar to the nominotypical subspecies *A. nigrizonatum* Hirano, 2021 in the adult (Fig. 1f), but differs markedly in the male and female genitalia (Figs 2d, 3b): in new subspecies, cucullus gently arcuate and evenly curved (in *A. nigrizonatum* strongly curved); costa with basal half of dorsal margin forming long, shallow concavity and distal half weakly sclerotised (in *A. nigrizonatum nigrizonatum* short, deep concavity and strongly sclerotised); sacculus process with anterior ridge connected to concavity (in *A. nigrizonatum* not connected); sacculus concavity narrowing strongly from outer to inner side (in *A. nigrizonatum*, gradually narrowing towards base). In female genitalia, ductus bursae straight and slightly sclerotised (in *A. nigrizonatum*, constricted, with prismatic longitudinal ridges); signum narrowly brush-shaped with fewer branches (in *A. nigrizonatum*, badminton shuttlecock-shaped, broader, with more branches).

#### Etymology

This subspecies is given its subspecific epithet according to its type locality, situated in China.

#### Distribution

China (Zhejiang, Yunnan, Fujian, Chongqing)

#### Biology

This subspecies has been recorded from montane forest regions in various parts of southern China, including Zhejiang, Yunnan, Fujian and Chongqing. Adults were collected by light trapping from July to September, indicating strong phototactic behaviour and suggesting that the adult activity period is mainly concentrated from late summer to early autumn. Its host plants and other ecological characteristics remain unknown.

#### Notes

The species is newly recorded from China. The nominotypical subspecies is restricted to Japan, whereas the new subspecies is distributed in southern China.

## Supplementary Material

XML Treatment for Araeopteron

XML Treatment for Araeopteron zheganensis

XML Treatment for Araeopteron nigrizonatum chinensis

## Figures and Tables

**Figure 1a.**
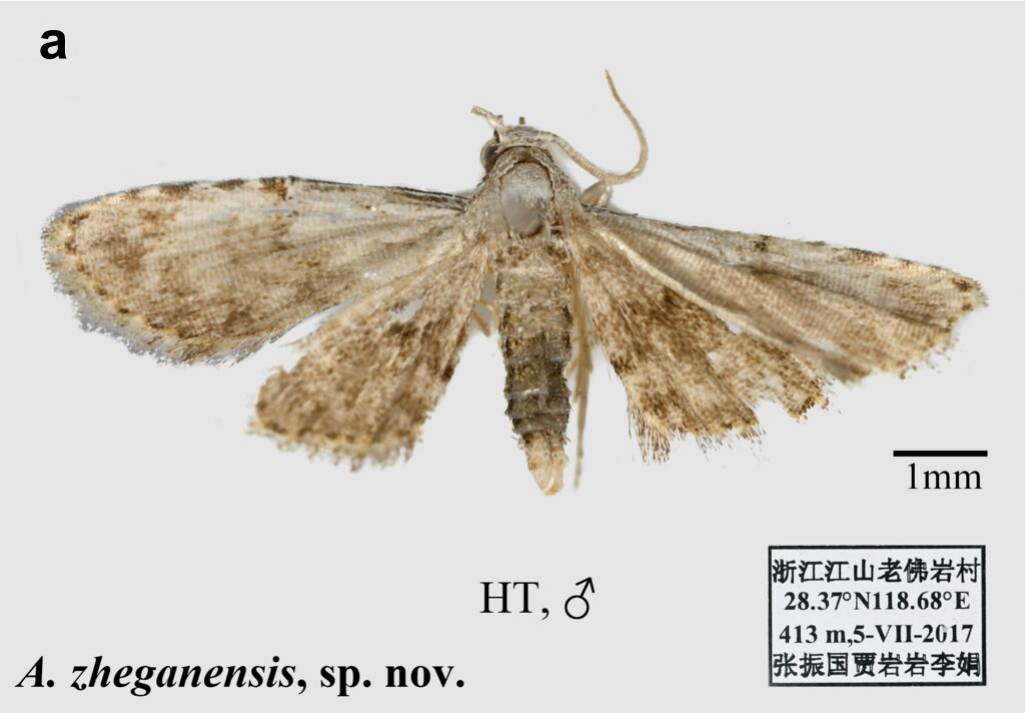
*A. zheganensis*, sp. nov., HT;

**Figure 1b.**
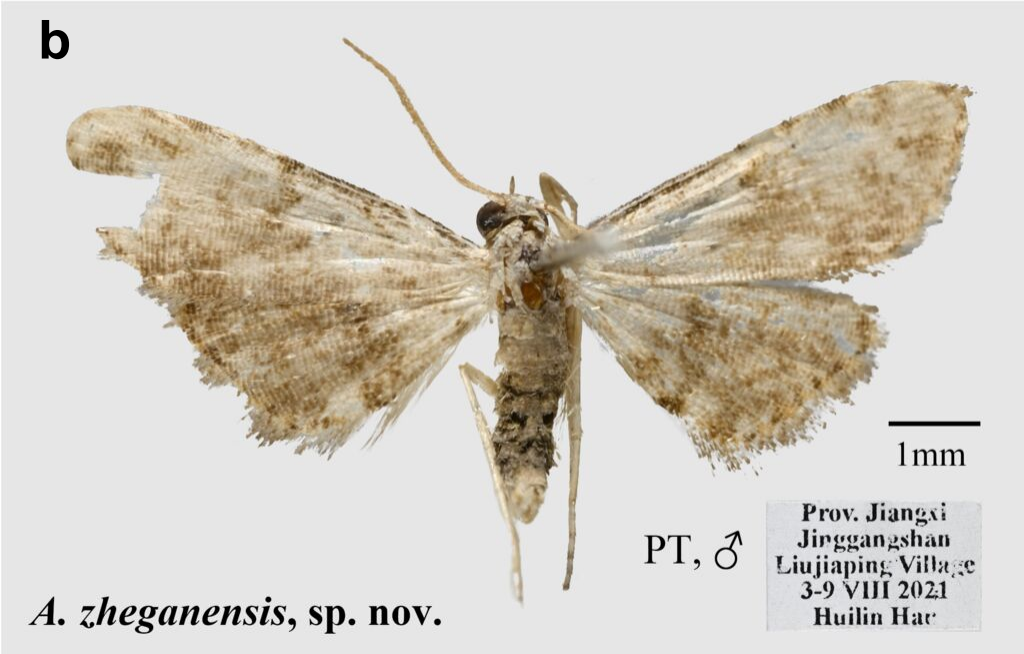
*A. zheganensis*, sp. nov., PT;

**Figure 1c.**
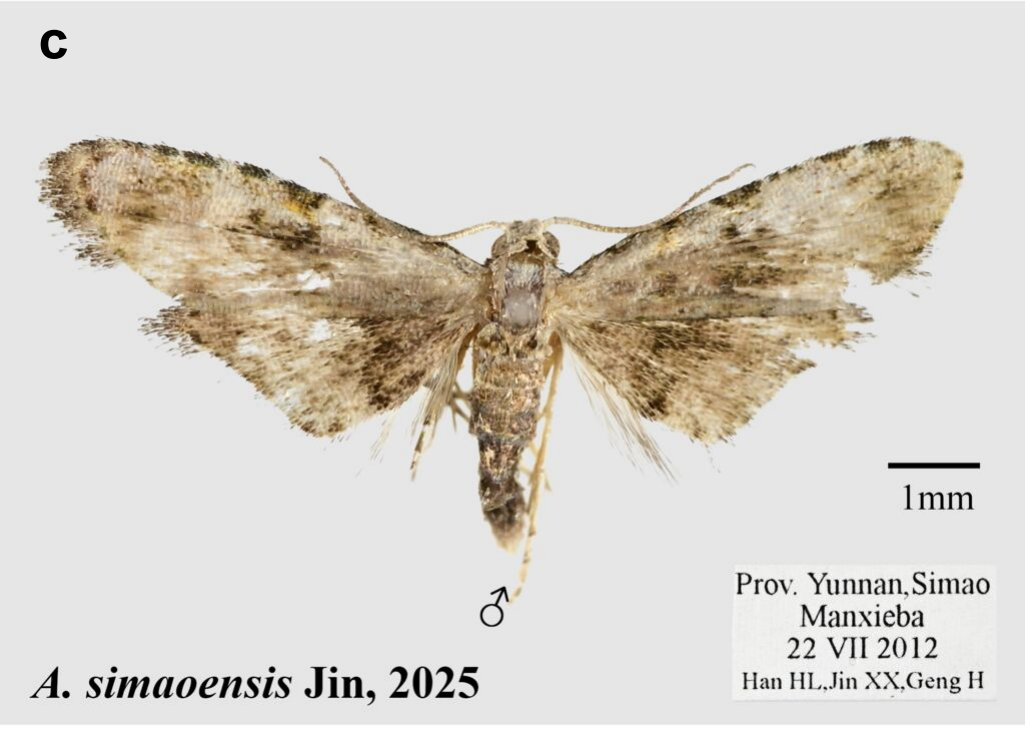
*A. simaoensis* Jin, 2025;

**Figure 1d.**
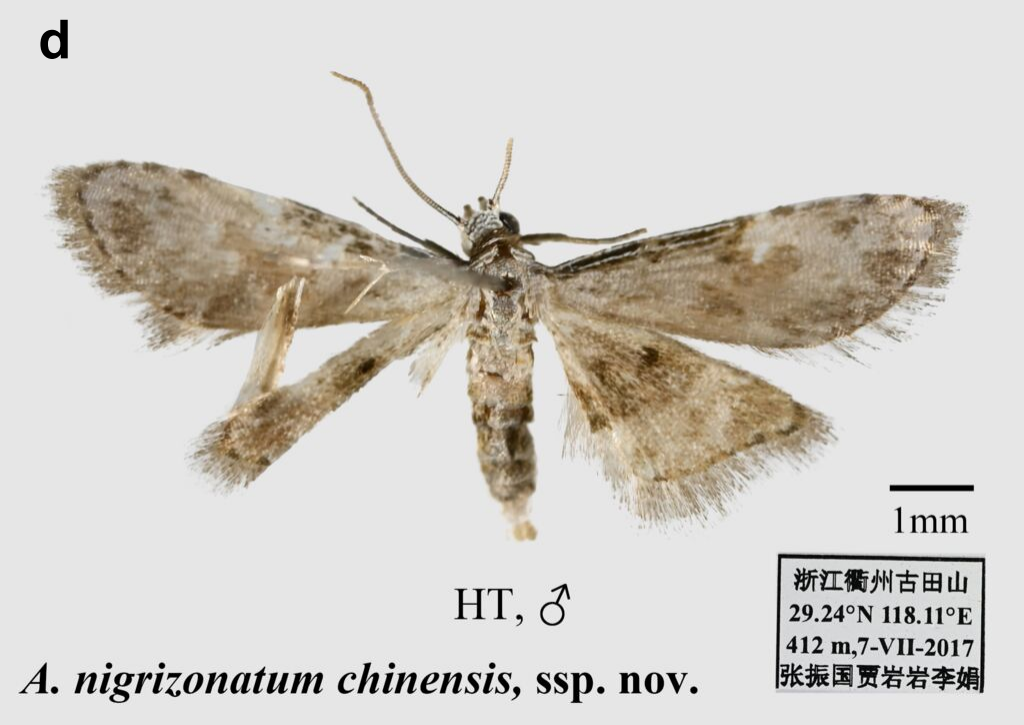
*A. nigrizonatum chinensis*, ssp. nov., HT;

**Figure 1e.**
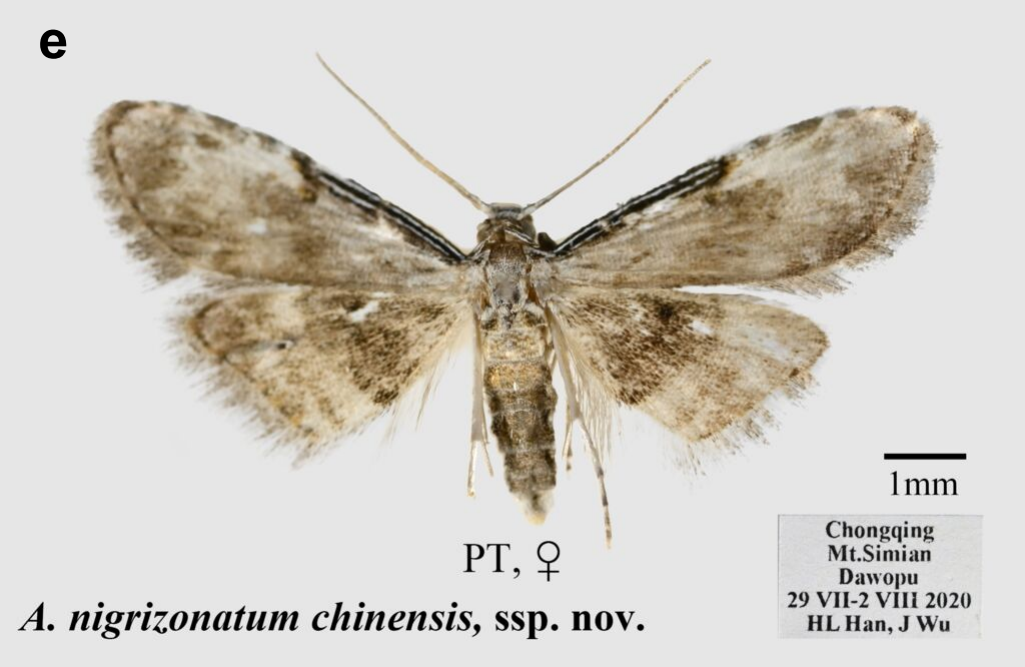
*A. nigrizonatum chinensis*, ssp. nov., PT;

**Figure 1f.**
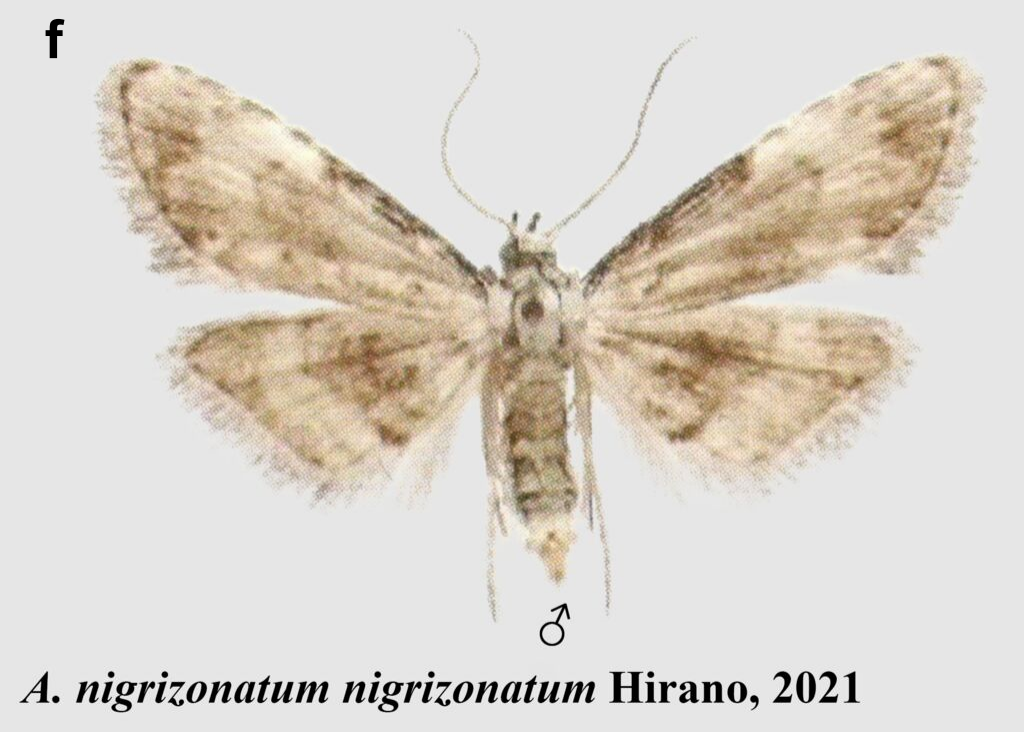
*A. nigrizonatum nigrizonatum* Hirano, 2021.

**Figure 2a.**
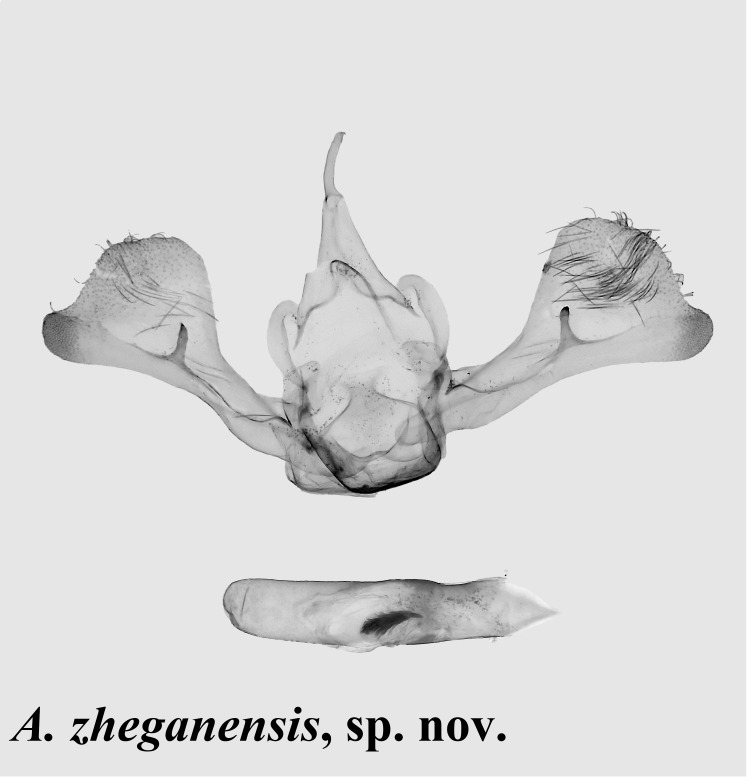
*A. zheganensis*, sp. nov., HT, slide YCH-434-1;

**Figure 2b.**
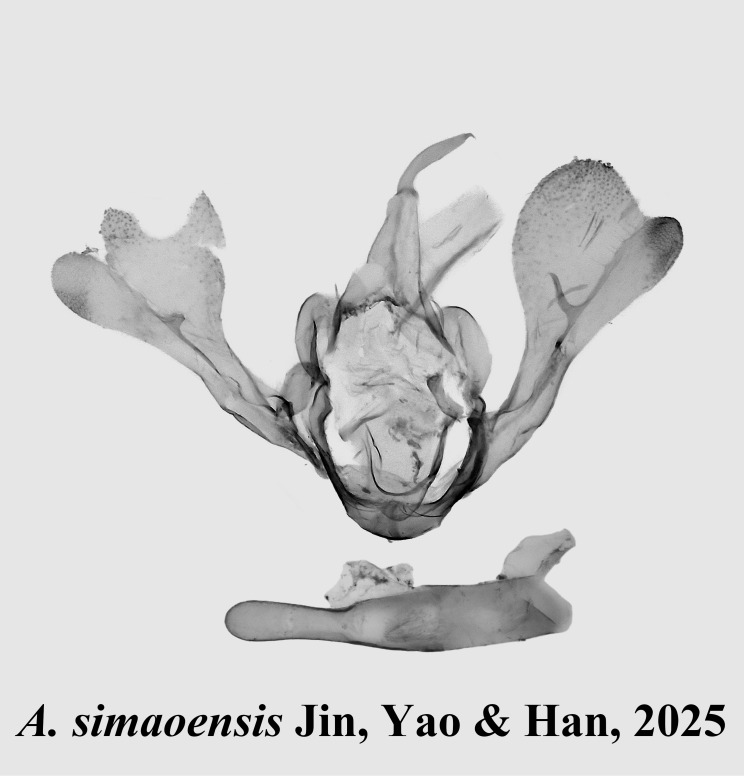
*A. simaoensis* Jin, Yao & Han, 2025, slide YCH-153-1;

**Figure 2c.**
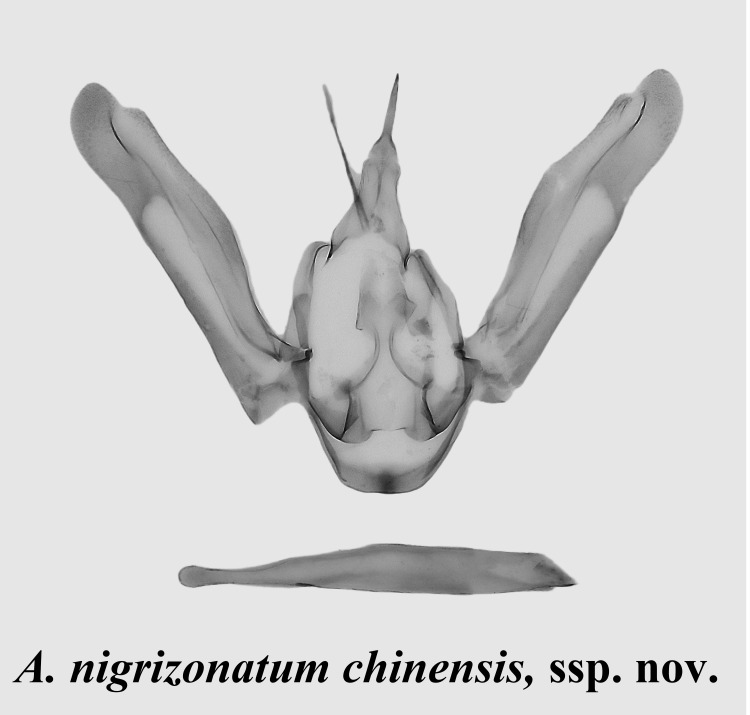
*A. nigrizonatum chinensis*, ssp. nov., HT, slide YCH-067-1;

**Figure 2d.**
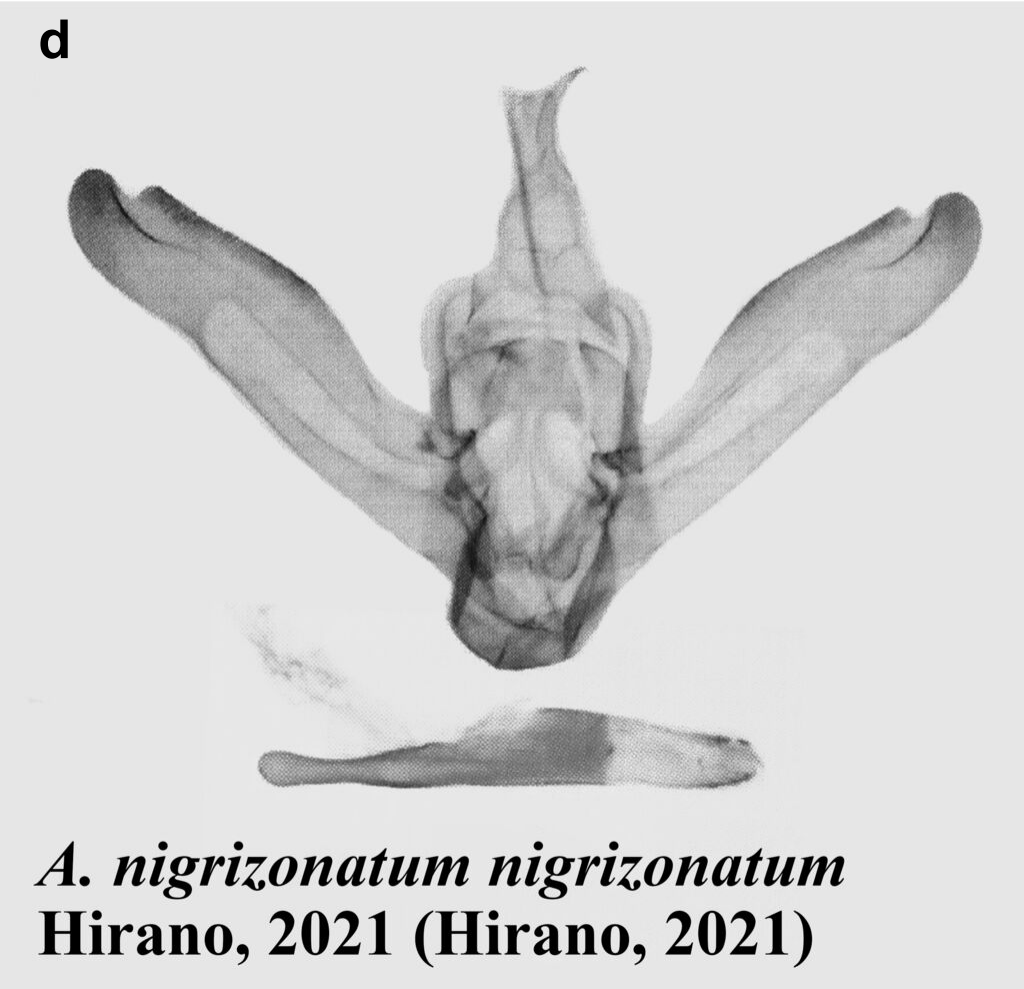
*A. nigrizonatum nigrizonatum* Hirano, 2021.

**Figure 3a.**
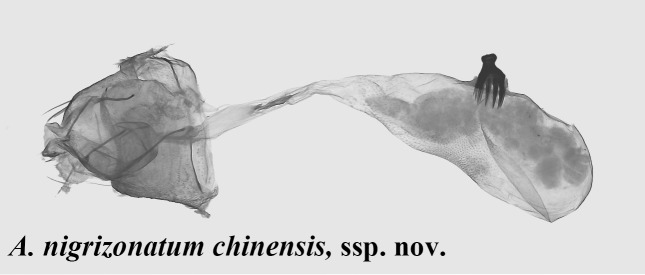
*A. nigrizonatum chinensis*, ssp. nov., PT, slide YCH-399-2;

**Figure 3b.**
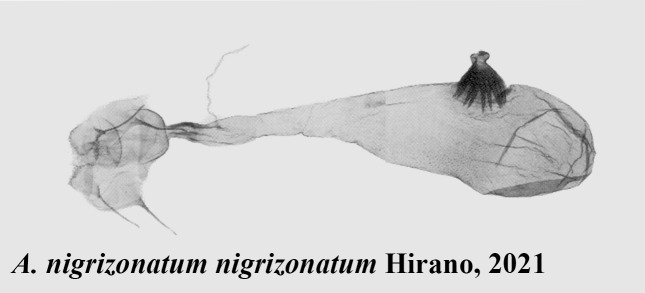
*A. nigrizonatum nigrizonatum* Hirano, 2021.
